# Photoaging Mobile Apps as a Novel Opportunity for Melanoma Prevention: Pilot Study

**DOI:** 10.2196/mhealth.8231

**Published:** 2017-07-26

**Authors:** Titus Josef Brinker, Dirk Schadendorf, Joachim Klode, Ioana Cosgarea, Alexander Rösch, Philipp Jansen, Ingo Stoffels, Benjamin Izar

**Affiliations:** ^1^ Department of Dermatology, Venerology and Allergology University-Hospital Essen University of Duisburg-Essen Essen Germany; ^2^ West German Cancer Center University of Duisburg-Essen Essen Germany; ^3^ German Cancer Consortium (DKTK) Heidelberg Germany; ^4^ Department of Medical Oncology Dana-Farber Cancer Institute Harvard Medical School Boston, MA United States; ^5^ Broad Institute of MIT and Harvard Cambridge, MA United States

**Keywords:** melanoma, skin cancer, prevention, mobile apps, smartphones, photoaging

## Abstract

**Background:**

Around 90% of melanomas are caused by ultraviolet (UV) exposure and are therefore eminently preventable. Unhealthy tanning behavior is mostly initiated in early adolescence, often with the belief that it increases attractiveness; the problems related to skin atrophy and malignant melanoma are too far in the future to fathom. Photoaging desktop programs, in which an image is altered to predict future appearance, have been successful in positively influencing behavior in adiposity or tobacco prevention settings.

**Objective:**

To develop and test a photoaging app designed for melanoma prevention.

**Methods:**

We harnessed the widespread availability of mobile phones and adolescents’ interest in appearance to develop a free mobile app called Sunface. This app has the user take a self-portrait (ie, a selfie), and then photoages the image based on Fitzpatrick skin type and individual UV protection behavior. Afterward, the app explains the visual results and aims at increasing self-competence on skin cancer prevention by providing guideline recommendations on sun protection and the ABCDE rule for melanoma self-detection. The underlying aging algorithms are based on publications showing UV-induced skin damage by outdoor as well as indoor tanning. To get a first impression on how well the app would be received in a young target group, we included a total sample of 25 students in our cross-sectional pilot study with a median age of 22 (range 19-25) years of both sexes (11/25, 44% female; 14/25, 56% male) attending the University of Essen in Germany.

**Results:**

The majority of enrolled students stated that they would download the app (22/25, 88%), that the intervention had the potential to motivate them to use sun protection (23/25, 92%) and that they thought such an app could change their perceptions that tanning makes you attractive (19/25, 76%). Only a minority of students disagreed or fully disagreed that they would download such an app (2/25, 8%) or that such an app could change their perceptions on tanning and attractiveness (4/25, 16%).

**Conclusions:**

Based on previous studies and the initial study results presented here, it is reasonable to speculate that the app may induce behavioral change in the target population. Further work is required to implement and examine the effectiveness of app-based photoaging interventions within risk groups from various cultural backgrounds.

## Introduction

Melanoma accounts for the majority of skin cancer-related deaths worldwide [1]. The implementation of next-generation sequencing has uncovered key oncogenic drivers of metastatic melanoma, such as mutations in the *BRAF* gene, which are present in around 50% of patients [2,3]. Development of therapies targeting the products of these genetic alterations, namely BRAF and MEK inhibitors, led to the first therapeutic revolution in melanoma care. These advances changed the prognosis of metastatic melanoma from a uniformly fatal disease with median survival of about 9 months to a treatable disease with median overall survival rates of more than 24 months, including some long-term responders [4-7].

In parallel, an improved understanding of mechanisms of tumor immune evasion, namely through interactions with immune checkpoints, such as cytotoxic T-lymphocyte-associated antigen 4 and programmed cell death protein 1, provided the rationale for the second therapeutic revolution. Conceptually, immune checkpoint inhibitors disrupt tumor-mediated T-cell dysfunction, and enable reactivation and effective immune-mediated tumor lysis. Up to 1 in 3 patients with metastatic melanoma may derive durable responses to these therapies [8-11].

Despite these disruptive changes in the therapeutic landscape, the majority of patients will still die from their disease. Several institutions and entities therefore emphasize and fund programs to improve preventive measures. Around 90% of melanomas are related to ultraviolet (UV) radiation [12], and recent data indicate that especially people with lower genetic risk for melanoma benefit from avoiding cumulative UV exposure, which is known as the divergent pathway hypothesis [13,14].

Unhealthy tanning behavior (including sunbed use) is mostly initiated in early adolescence [15], often with the belief that it increases attractiveness [16-18]; the problems related to skin atrophy and malignant melanoma are too far in the future to fathom.

To target populations at risk for such behaviors, implementation of programs that are embedded in frequently used media, such as the Internet or mobile phone apps, may be useful. Indeed, a recent randomized controlled trial by Burford et al demonstrated the effectiveness of photoaging desktop programs, in which an image is altered to predict future appearance, for behavioral change in young target groups in the field of smoking cessation [19]. Furthermore, a quasi-experimental study showed significantly higher scores for predictors of sun protection behavior in young women from the United Kingdom using such programs [20], which were also effective in changing young adults’ suntanning intentions in both sexes [21]. However, the investigated desktop-based programs only reach a small audience and are not freely available. To improve melanoma prevention in the larger population by leveraging frequently used technologies, we developed a freely available phone app aimed at enhancing sun protective behaviors.

## Methods

**Figure 1:**
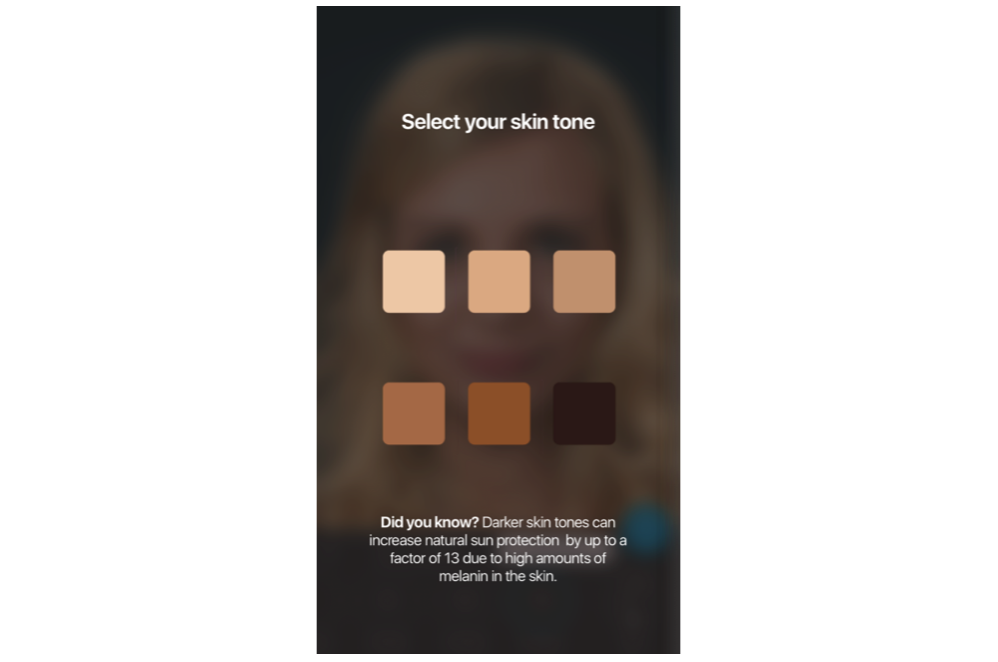
Start of the app: the user picks their Fitzpatrick skin type.

**Figure 2:**
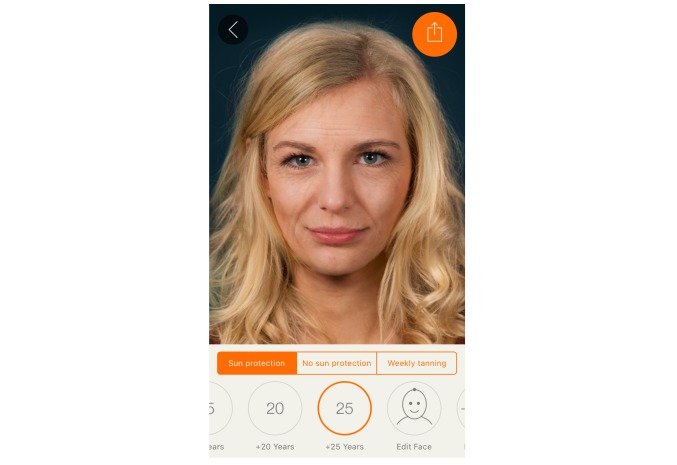
Effect view of the app: 25 years of aging with applied sun protection.

**Figure 3:**
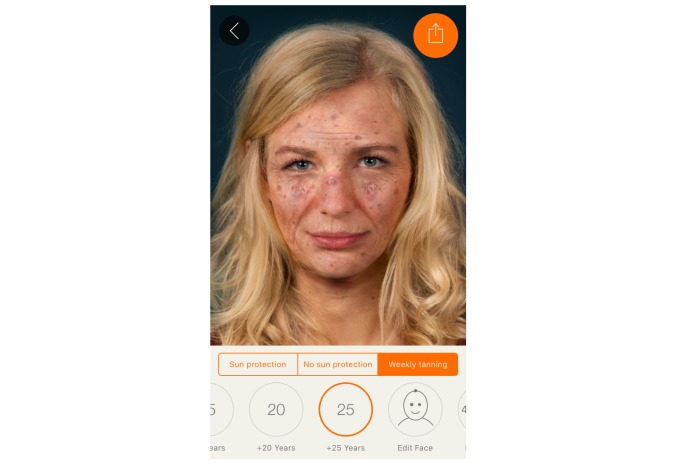
Effect view of the app: weekly tanning for 25 years (maximum effect) with a total of 3 actinic keratoses visible, multiple solar lentigines, age spots, and prominent solar elastosis.

**Figure 4:**
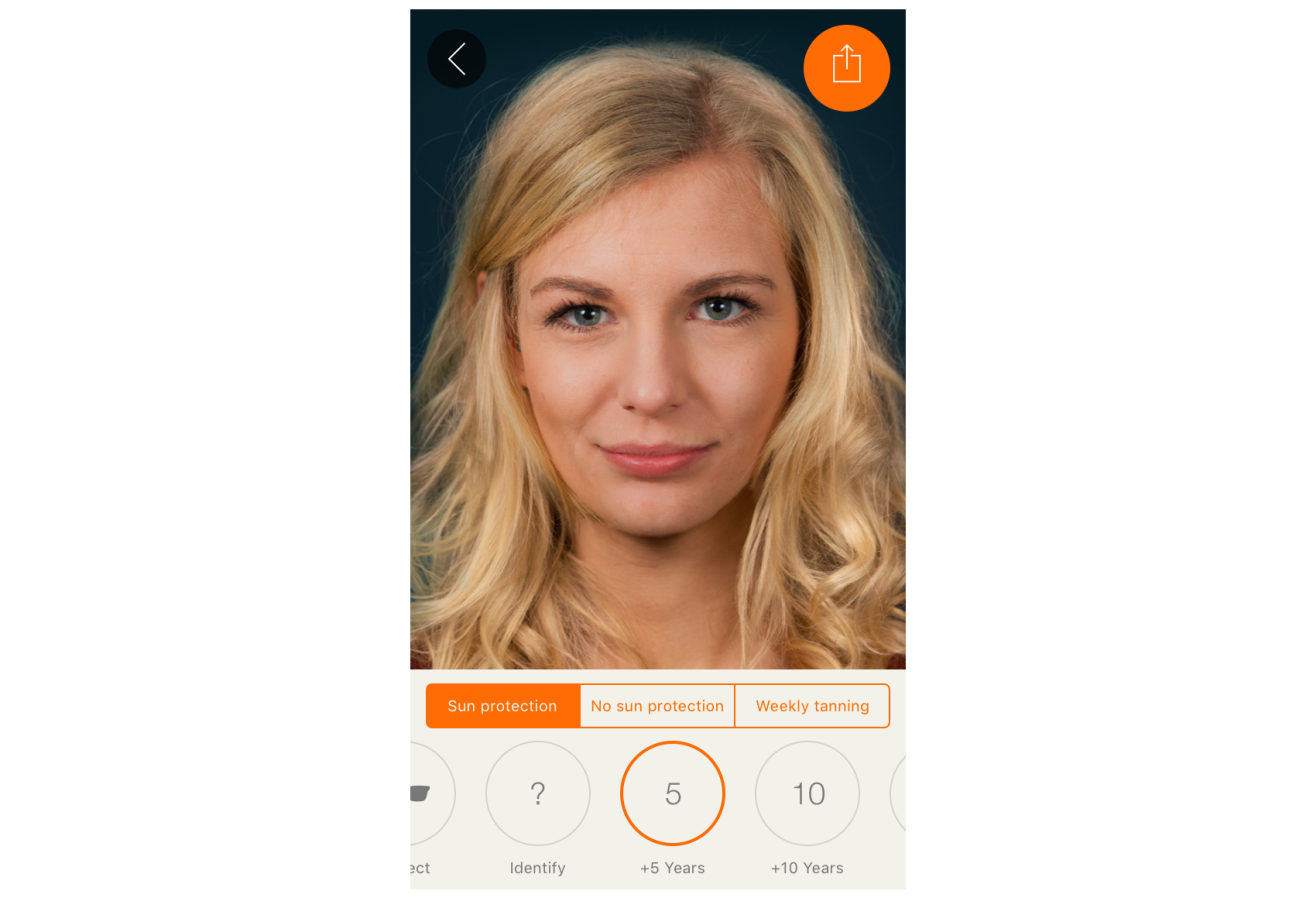
Effect view of the app: 5 years of normal aging with daily sun protection applied.

We harnessed the widespread availability of mobile phones and adolescents’ interest in appearance to develop a free mobile app. The Sunface app has the user take a self-portrait (ie, a selfie), and then photoages the image based on Fitzpatrick skin type (Figure 1) and individual UV protection behavior (Figure 2, Figure 3, Figure 4).

**Figure 5:**
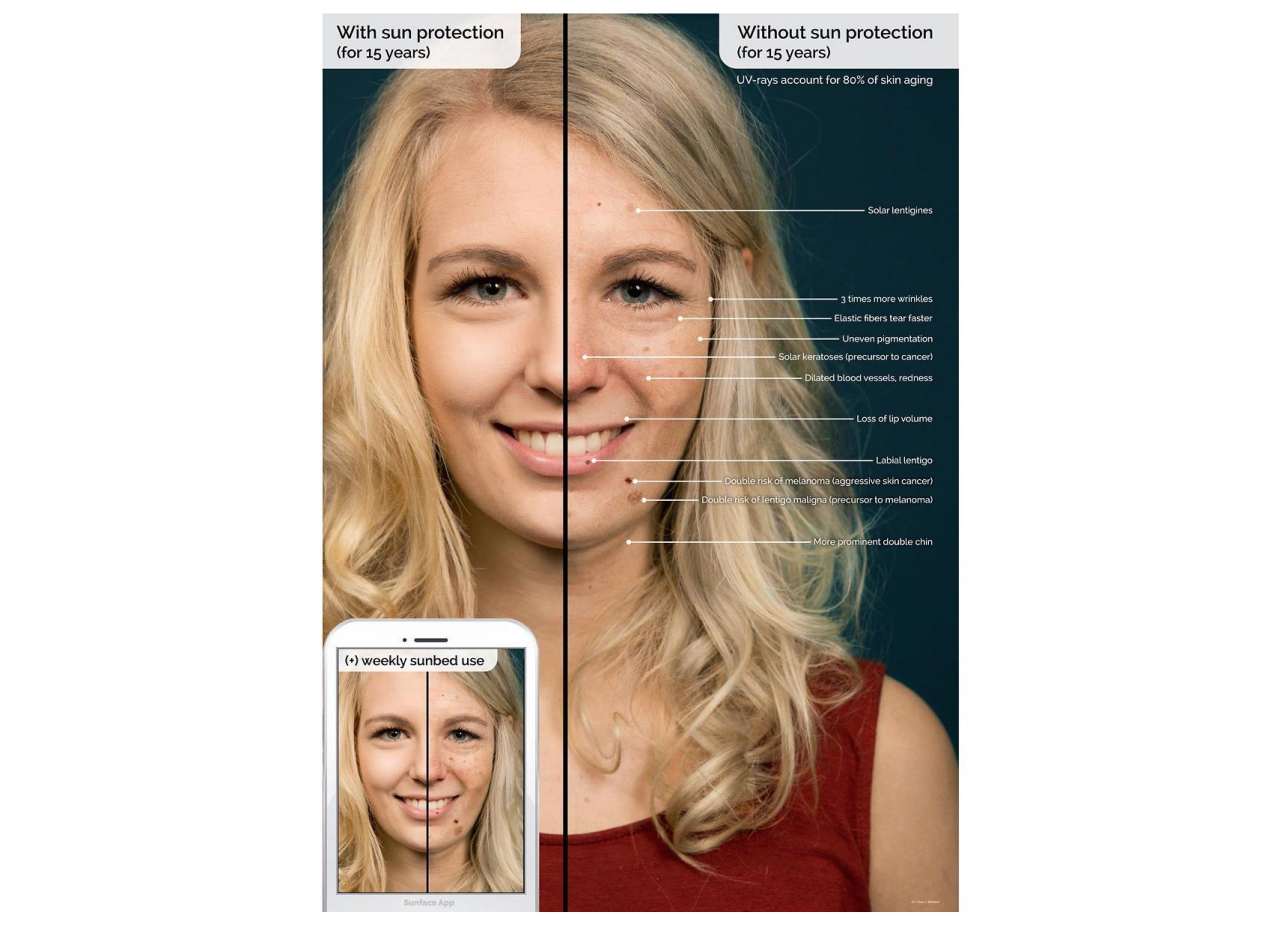
Explanatory graphic within the app explaining the shown effects. UV: ultraviolet.

Afterward, the app explains the visual results (Figure 5) and increases self-competence on skin cancer prevention by providing guideline recommendations on sun protection and the ABCDE rule for melanoma self-detection (assess border irregularity, color variety, diameter, and evolution [22]). By means of sharing tools of the animated image as a video (Multimedia Appendix 1) or photo, the user’s social network may be informed about the various beauty-reducing effects of tanning and about the app.

The underlying aging algorithms are based on publications showing UV-induced skin damage caused by outdoor as well as indoor tanning [23]. As no trials with 25 years of follow-up were available, we had to extrapolate the current evidence on UV-induced skin damage for the specific skin types.

To get a first impression of how well the app would be received in a young target group, we included a total sample of 25 students in our cross-sectional pilot study with a median age of 22 (range 19-25) years of both sexes (11/25, 44% female; 14/25, 56% male) attending the University of Essen in Germany.

An interviewer walked up to each individual student, asked for oral consent, let them use the app once by handing them an iPod Touch (Apple Inc) with the app preinstalled, and then measured their reactions to the 1-time use of the app (no longer than 2 minutes) via a paper-and-pencil questionnaire. The items used in the questionnaire captured sociodemographic data (sex, age) and their reactions toward the app, on 5-point Likert scales, directly after using it. All items used wording and a structure similar to those of a previously published questionnaire evaluating a photoaging app for tobacco use prevention [24]. Each selfie was deleted directly after the individual test persons had tested the app for data protection reasons.

## Results

The majority of enrolled students stated that they would download the app (22/25, 88%), that the intervention had the potential to motivate them to use sun protection (23/25, 92%), and that they thought such an app could change their perceptions that tanning makes you attractive (19/25, 76%). Only a minority of students disagreed or fully disagreed that they would download such an app (2/25, 8%) or that such an app could change their perceptions on tanning and attractiveness (4/25, 16%).

In line with our small survey, the app was installed on over 1000 Android and 500 iOS smartphones within 14 days after its release in Germany (May 30, 2017 to June 17, 2017). We thus expect it to reach a similar popularity with an estimated 30,000 users within 1 year, which is comparable with our photoaging app on tobacco-induced skin changes [25]. As smartphone use in Germany declines with age, we assume that the largest fraction of app users will be in the vulnerable age group of 35 years or younger.

## Discussion

The implementation of novel technologies and computational algorithms has the potential to substantially change the landscape of cancer prevention and early melanoma detection [19,20,24,26-30], and thereby reduce its disease-specific mortality. Here, we propose the use of a mobile phone app, Sunface, as a means to implement interventions that encourage sun protective behavior. The effectiveness of such approaches has been demonstrated in recent studies, and could be complimentary to early detection programs by dermatologists and recently developed artificial intelligence programs [31]. Phone apps in the field of dermatology are of increasing relevance [32-44] and may be particularly effective in reaching a large number of people, given the increasing use of mobile phones, which is projected to increase to more than 6 billion subscriptions by 2021 [45], and their integration into daily living habits by these customers.

Based on previous studies and the initial study results presented here, it is reasonable to speculate that the app may induce behavioral change in the target population. Further work is required to implement and examine the effectiveness of app-based photoaging interventions within risk groups from various cultural backgrounds.

## Appendix

Multimedia Appendix 1 Shared video of the Sunface app with 15 years of no sun protection shown in a 3-dimensional animated selfie.

## Abbreviations

UV: ultraviolet
